# A Broad-Spectrum Antiviral Molecule, Protoporphyrin IX, Acts as a Moderator of HIV-1 Capsid Assembly by Targeting the Capsid Hexamer

**DOI:** 10.1128/spectrum.02663-22

**Published:** 2022-12-07

**Authors:** Da-Wei Zhang, Liangxu Xie, Xiao-Shuang Xu, Yimin Li, Xiaojun Xu

**Affiliations:** a Institute of Bioinformatics and Medical Engineering, School of Electrical and Information Engineering, Jiangsu University of Technology, Changzhou, China; b College of Pharmacy and Key Laboratory for Research and Development of “Qin Medicine” of Shaanxi Administration of Chinese Medicine, Shaanxi University of Chinese Medicine, Xixian New District, China; Kumamoto University

**Keywords:** HIV-1 capsid, capsid assembly, hexamer, drug screening, biolayer interferometry

## Abstract

The capsid protein (CA), an essential component of human immunodeficiency virus type 1 (HIV-1), represents an appealing target for antivirals. Small molecules targeting the CAI-binding cavity in the C-terminal domain of HIV-1 CA (CA CTD) confer potent antiviral activities. In this study, we report that a small molecule, protoporphyrin IX (PPIX), targets the HIV-1 CA by binding to this pocket. PPIX was identified via *in vitro* drug screening, using a homogeneous and time-resolved fluorescence-based assay. CA multimerization and a biolayer interferometry (BLI) assay showed that PPIX promoted CA multimerization and bound directly to CA. The binding model of PPIX to CA CTD revealed that PPIX forms hydrogen bonds with the L211and E212 residues in the CA CTD. Moreover, the BLI assay demonstrated that this compound preferentially binds to the CA hexamer versus the monomer. The superposition of the CAI CTD-PPIX complex and the hexameric CA structure suggests that PPIX binds to the interface formed by the NTD and the CTD between adjacent protomers in the CA hexamer via the T72 and E212 residues, serving as a glue to enhance the multimerization of CA. Taken together, our studies demonstrate that PPIX, a hexamer-targeted CA assembly enhancer, should be a new chemical probe for the discovery of modulators of the HIV-1 capsid assembly.

**IMPORTANCE** CA and its assembled viral core play essential roles in distinct steps during HIV-1 replication, including reverse transcription, integration, nuclear entry, virus assembly, and maturation through CA–CA or CA–host factor interactions. These functions of CA are fundamental for HIV-1 pathogenesis, making it an appealing target for antiviral therapy. In the present study, we identified protoporphyrin IX (PPIX) as a candidate CA modulator that can promote CA assembly and prefers binding the CA hexamer versus the monomer. PPIX, like a glue, bound at the interfaces between CA subunits to accelerate CA multimerization. Therefore, PPIX could be used as a new lead for a CA modulator, and it holds potential research applications.

## INTRODUCTION

Although enormous efforts have been made to prevent the transmission of human immunodeficiency virus type 1 (HIV-1), the etiological agent of acquired immunodeficiency syndrome (AIDS), AIDS-related deaths and new infections remain a global public health problem (1). Combination antiretroviral therapy (cART) can effectively abolish HIV-1 replication and restrict disease progression to AIDS. However, cART does not eradicate HIV-1, and patients need lifelong therapy, which may cause side effects and HIV drug resistance (2, 3). Consequently, strategies for development of new antiretroviral therapies are necessary to contain the AIDS epidemic and even cure the disease.

The mature HIV-1 particle has a cone-shaped shell that is enveloped by a lipid-rich membrane (4–6). The proper formation and integrity of the virus core is critical during HIV-1 infection (7). The viral core is built up by approximately 1,500 capsid proteins (CA, p24). These building blocks form 250 hexamers and 12 CA pentamers and are rearranged into a fullerene-shaped virus core (4). CA and its assembled viral core play essential roles in distinct steps during HIV-1 replication, including reverse transcription, integration, nuclear entry, virus assembly, and maturation through CA–CA or CA–host factor interactions (8, 9). These functions of CA are fundamental for HIV-1 pathogenesis, making it an appealing target for antiviral therapy (10, 11).

Over the past 20 years, numerous CA-targeting small molecules have been reported (reviewed in references [12, 13]). Among them, a long-acting drug GS-6207 (lenacapavir) is the most successful one, and it is currently being evaluated in ongoing phase II and III clinical studies (14). These compounds target five distinct binding sites in CA (12): the FG (phenylalanine-glycine) binding site, exemplified by PF74, GS-6207, BI-1, BMMP, and C-A1 (15–18); the N-(3-chloro-4-methylphenyl)-N0-{2-[({5-[(dimethylamino)-methyl]-2-furyl}-methyl)-sulfanyl]ethyl}urea) binding site, interacting with BD3, BM4, and CAP-1 (19, 20); the 2-fold binding site, targeted by CAI, I-XW-053, compound 16, ebselen, and MKN-1A (21–25); the apical binding site, interacting with some benzimidazole compounds (26); and the central pore binding site, targeted by ligands HCB and ACAi-028 (27, 28).

Monomeric CA consists of a helical N-terminal domain (CA_NTD_) and a C-terminal domain (CA_CTD_), that are connected by a short, flexible linker (29). The binding pockets of most described CA-targeting compounds are mapped onto CA_NTD,_ but only a few compounds, such as CAI, ebselen, and MKN-1A, bind to CA_CTD_. CAI, a 12-mer peptide, blocks the assembly of immature HIV-1 particles as well as that of mature HIV-1 particles *in vitro* (21). The structure of the CAI-CA CTD complex reveals that CAI allosterically modifies the CA CTD dimer interface by binding to a conserved cavity that is formed by three α helices (α8, α9, and α11) of CA CTD (30, 31). Thereafter, several compounds, including stapled peptides and small molecules, are described to bind to this conserved pocket, showing their potent activity against the replication of HIV-1 in cell cultures (22, 32–34). Collectively, these compounds validate that the CAI-binding pocket can be used as a target for antiviral development.

In the present study, we sought to obtain new chemicals that are capable of binding to the CAI-binding cavity by screening a commercially available drug library via a homogeneous time-resolved fluorescence (HTRF) assay. We identified protoporphyrin IX (PPIX) as a candidate CA modulator that can promote CA assembly and prefers binding the CA hexamer versus the monomer. It is anticipated that PPIX, like a glue, binds at the interfaces between CA subunits to accelerate CA multimerization. Therefore, PPIX could be used as a new lead for a CA modulator, and it holds potential research applications.

## RESULTS

### Identification of candidate drugs targeting the CAI-binding pocket.

**FIG 1:**
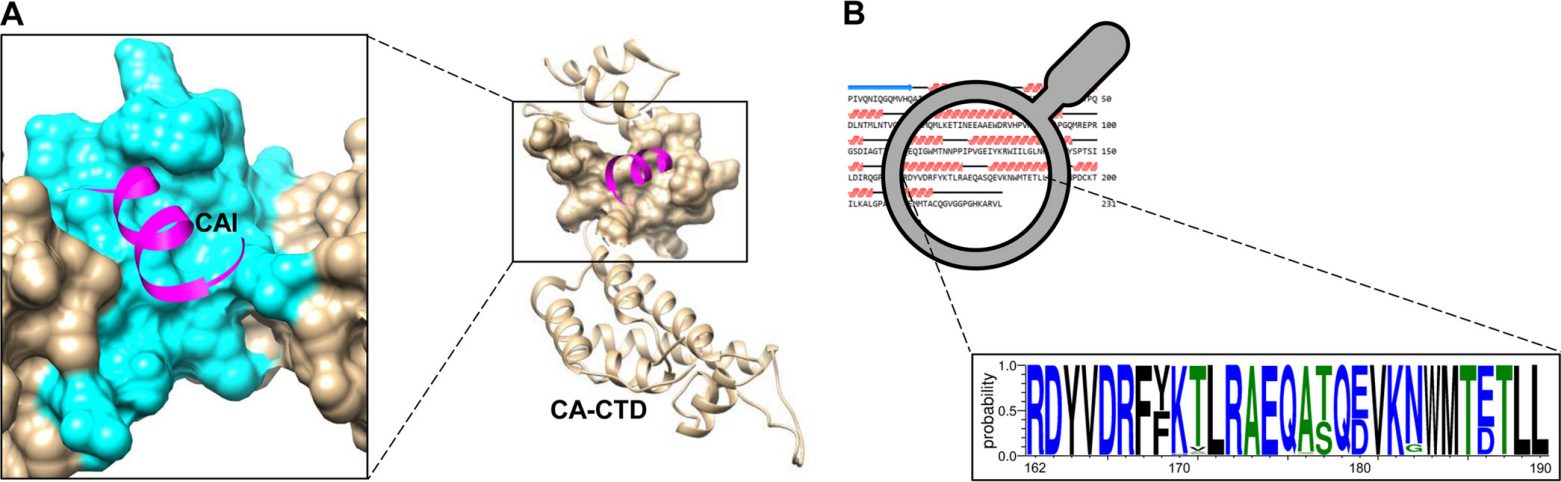
Profile of CA CTD and its CAI-binding pocket. (A) The crystal structure of CA CTD (PDB ID: 2BUO). The groove bound by the CAI peptide (magenta) is highlighted in cyan. The images were created with UCSF Chimera. (B) Residues from 162 to 190 of CA CTD, shown as a WebLogo, using the WebLogo (version 3.7.4). These residues mainly constitute the CAI-binding cavity and are highly conserved.

**FIG 2:**
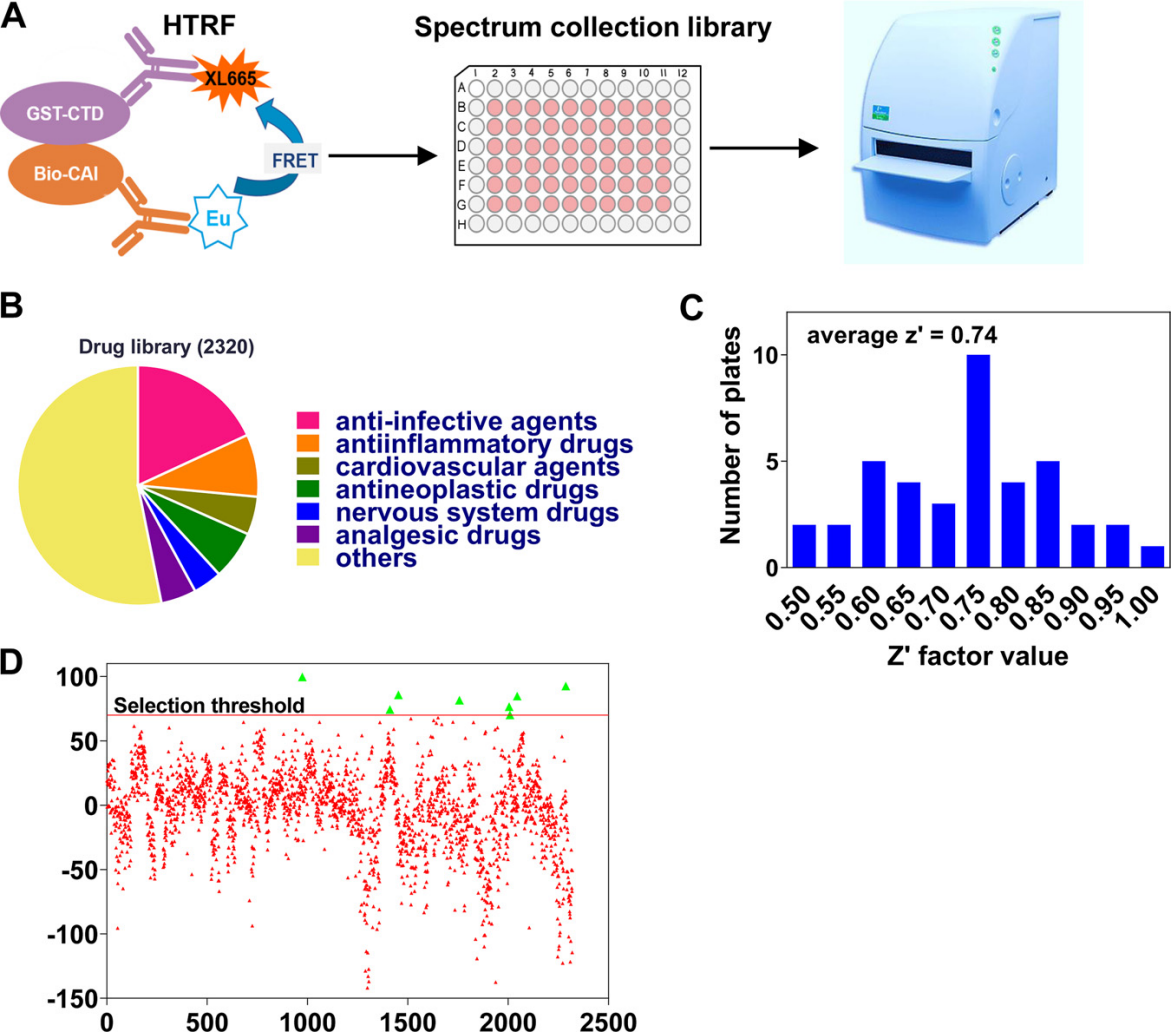
Primary screening for inhibitors of CA CTD-CAI interaction. (A) Flowchart of *in vitro* screening to identify the candidates targeting the CAI-binding pocket in CA CTD. (B) Composition of the compound library used in the screen. (C) Z′ factor frequency distribution for 29 screening plates. The average value of the Z′ factor was 0.74. (D) Drug screening for primary candidates of inhibitors of CA CTD-CAI interaction from the Spectrum Collection Library at 50 μM. The inhibition rates of all of the compounds used in the primary screening are presented as scattered points. The green triangle indicates 8 compounds with inhibition rates of >70% that were selected for a secondary assay.

The binding of dodecapeptide CAI to a hydrophobic cavity (Fig. 1A) in CA CTD allosterically locks CA CTD in a nonfunctional conformation, leading to the blockage of immature and mature virus assembly *in vitro*. Most of the CA residues bound by CAI are mapped to the region between residues 162 and 190, which are highly conserved (Fig. 1B). To identify novel capsid inhibitors, this conserved pocket was selected as a potential site targeted by candidate compounds. The screening strategy and process are summarized in Fig. 2A. We conducted the screen using the Spectrum Collection library. The library consists of 2,320 small molecules that have been used for treating infectious diseases, neurodegenerative disorders, psychiatric disorders, cardiovascular diseases, and cancer (Fig. 2B). These compounds were tested at 50 μM in two replicates. Compound information, including names, bioactivity, CAS numbers, sources, references, and percent inhibition values for both screening replicates are presented in Table S1 and Table S2. The assays showed an excellent Z′ value (an indicator for assessing the quality of drug screening) of 0.74 ± 0.13 in the primary screening, using 29 different plates (Fig. 2C). Eight compounds were identified as “hits”, meaning that they caused the statistically significant inhibition (>70%) of CA CTD–CAI interaction (Fig. 2D; Table 1).

**TABLE 1:** Hits obtained from the primary screening of the Spectrum Collection Library for the disruption of the CA CTD–CAI interaction

Compound	Percentage inhibition (50 μM)
Etylpyridinium chloride	92.64
Riboflavin	99.63
Protoporphyrin IX	74.25
Thioctic acid	85.75
Dihydrogambogic acid	78.11
Stictic acid	81.58
Norstictic acid	70.07
Theaflavin	76.09

### PPIX shows a dose-dependent inhibitory effect on CA CTD-CAI interaction and binds directly to CA.

**FIG 3:**
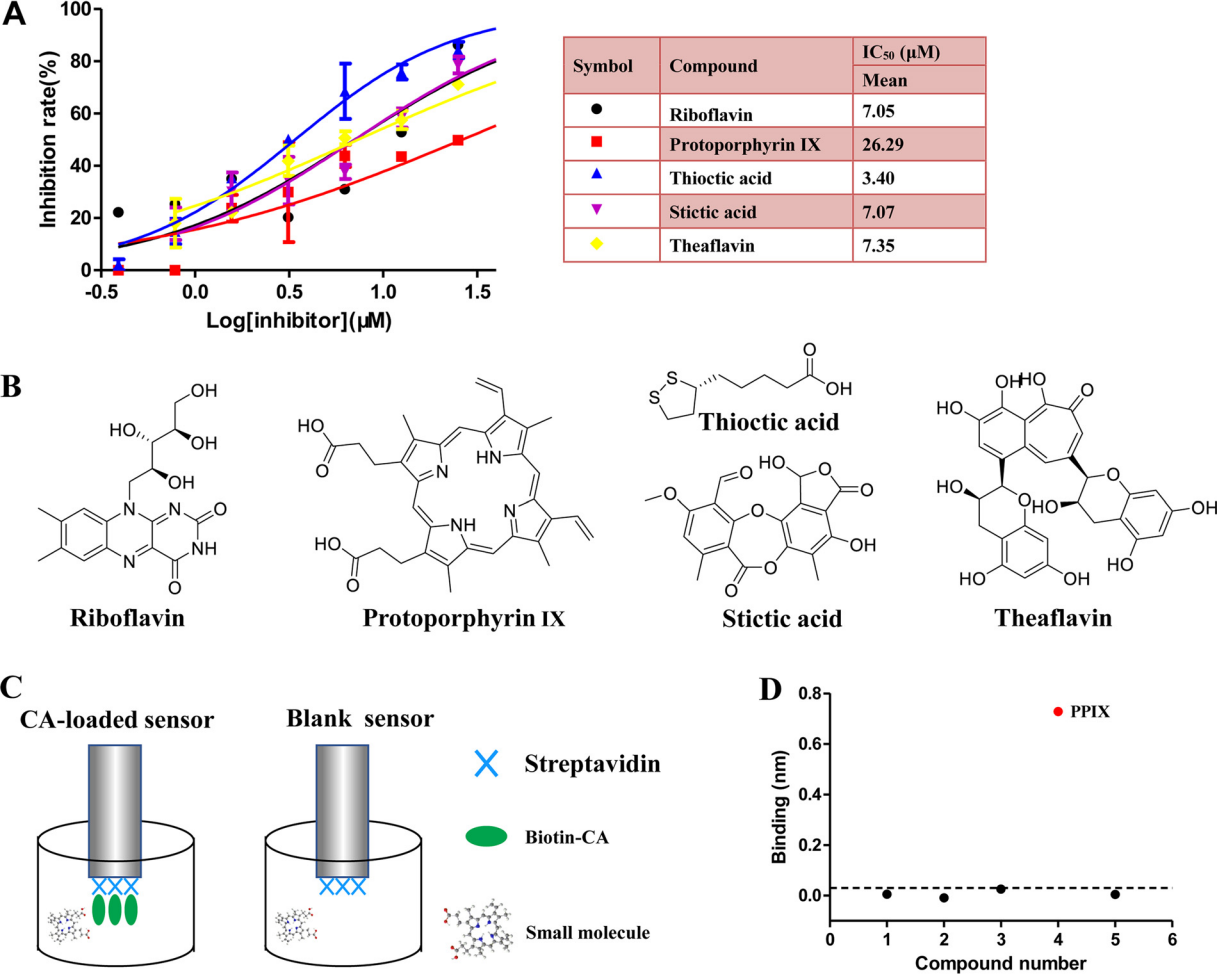
PPIX interacts directly with CA and inhibits CA CTD-CAI interaction in a dose-dependent fashion. (A) Dose-response curves of riboflavin (black), protoporphyrin IX (red), thioctic acid (blue), stictic acid (purple), and theaflavin (yellow). (B) Chemical structures of compounds showing the dose-dependent inhibition of CA CTD-CAI interaction. (C) Schematic illustration of the double reference subtracted analysis via biolayer interferometry (BLI). (D) The responses of the compounds screened at a single concentration of 30 μM. PPIX with responses of >0.03 nm are labeled in red.

The potency of 8 hits against CA CTD–CAI interaction was confirmed in a dose-response assessment over a concentration range from 0.39 μM to 50 μM, using identical conditions in a HTRF assay. Of the 8 hits, 5 compounds showed a dose-dependent inhibition of the interaction (Fig. 3A). Among the 5 compounds, thioctic acid had the greatest inhibitory effect, displaying an IC_50_ of 3.4 μM, which is 9-fold better than that of protoporphyrin IX (PPIX). Three hits (riboflavin, stictic acid, and theaflavin) showed modest IC_50_ values of 7.0 μM. The structures of the 5 hits are depicted in Fig. 3B. Then, these five compounds were analyzed by the double reference subtracted BLI assay (as depicted in Fig. 3C) to confirm their affinity to CA. The assay was performed with a single compound concentration of 30 μM for its binding to monomeric CA. Surprisingly, only PPIX exhibited a relatively high response above 0.03 nm, whereas the other four compounds showed negligible binding to the CA monomer (Fig. 3D). Moreover, we detected the affinity of these five compounds to the CA hexamer. As shown by the results in Fig. S1, PPIX exhibited its potency in binding to the CA hexamer, whereas the other four compounds showed negligible binding to the CA hexamer. These results demonstrated that PPIX directly bound to CA as a competitor of the CAI-CA CTD interaction. Thus, PPIX was selected for subsequent analysis.

### PPIX increases CA multimerization *in vitro*.

**FIG 4:**
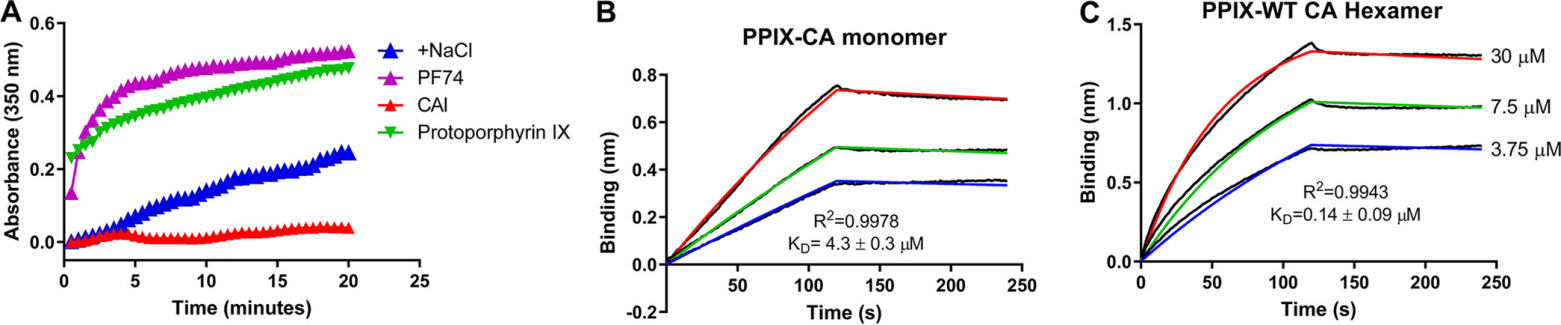
PPIX induces CA multimerization and preferentially binds to the CA hexamer. (A) Kinetics of CA multimerization under high salt (2.5 M NaCl) conditions, with or without protoporphyrin IX. The results are representative of two independent experiments. The binding of PPIX to monomeric CA (B) and hexameric CA (C), as analyzed via BLI. All of the Sensorgrams were fit using OctetRed user software (version 9.2) with a global 1:1 model to obtain the values of the three constants k_on_, k_off_, and K_D_.

*In vitro*, HIV-1 CA spontaneously assembles under conditions of high ionic strength into open-ended, helical tubes, which causes a change in optical density. The ability of PPIX to disrupt CA tube assembly was monitored by a turbidity-based HIV-1 CA assembly assay with or without the compound (Fig. 4A). PF74 and CAI were included as controls. As expected, the addition of PF74 (purple) led to a strong increase of CA multimerization, whereas the addition of the CAI peptide (red) almost completely abolished CA assembly. Interestingly, PPIX (green), which interrupted the CA CTD-CAI interaction as did CAI, accelerated *in vitro* CA assembly as did PF74. These data demotrated that PPIX binding disrupts capsid assembly *in vitro*.

### PPIX preferentially binds to the hexameric form of CA.

PPIX was further tested with a series of concentrations for its affinity to the CA monomer and hexamer. PPIX bound to the CA hexamer with a K_D_ of 0.53 μM (Fig. 4B) and bound to the CA monomer with a K_D_ of 4.30 μM (Fig. 4C). PPIX showed a more than 8-fold higher-affinity when binding to the CA hexamer, relative to the CA monomer.

**FIG 5:**
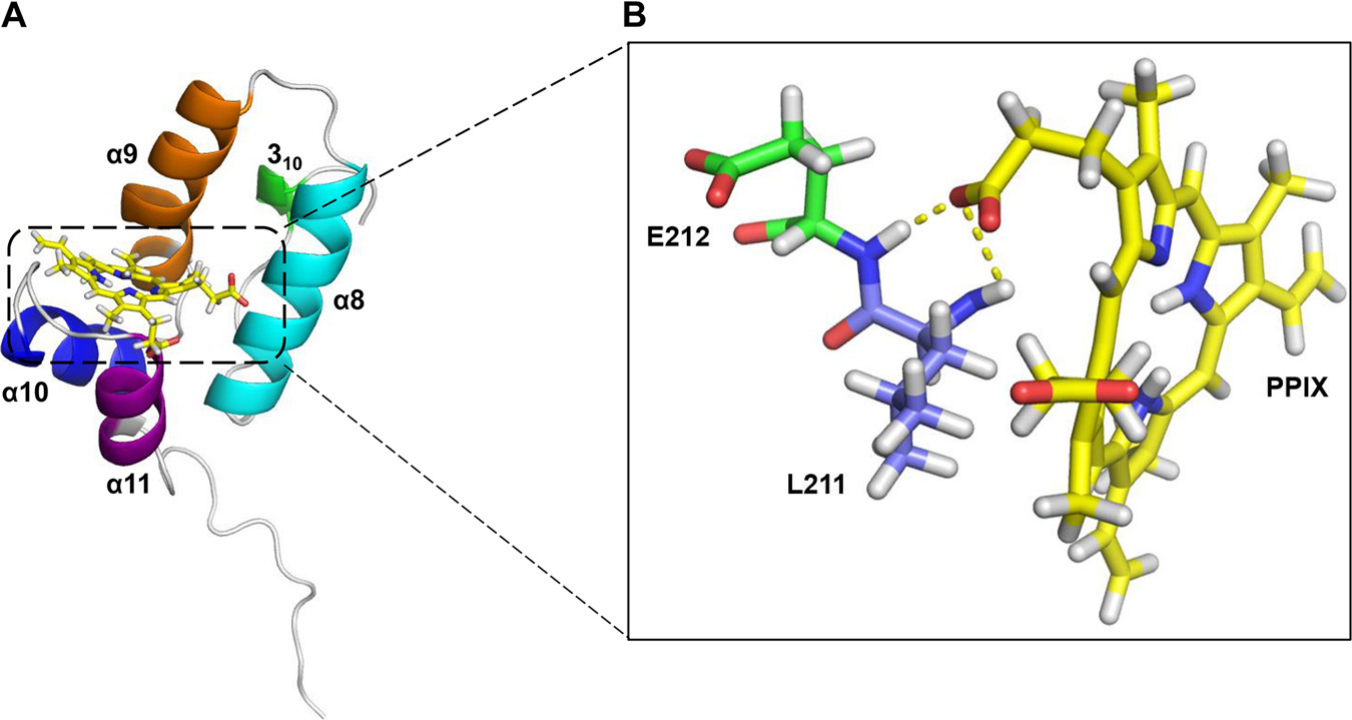
The binding mode PPIX with CA CTD. (A) The docking result of PPIX with CA CTD. The CA CTD is shown as a cartoon. The four 5-helices (3_10_, α8, α9, α10, α11) are highlighted in green, cyan, orange, blue, and purple. PPIX is shown as sticks (C in yellow, H in white, N in blue, and O in red). Molecular docking was performed with Swiss-Dock. (B) Details of key residues interacting with PPIX. The hydrogen bonds between GS-6207 and the CA residues (L211 and E212) are shown as dashed yellow lines. All molecular graphics were generated using PyMOL.

**FIG 6:**
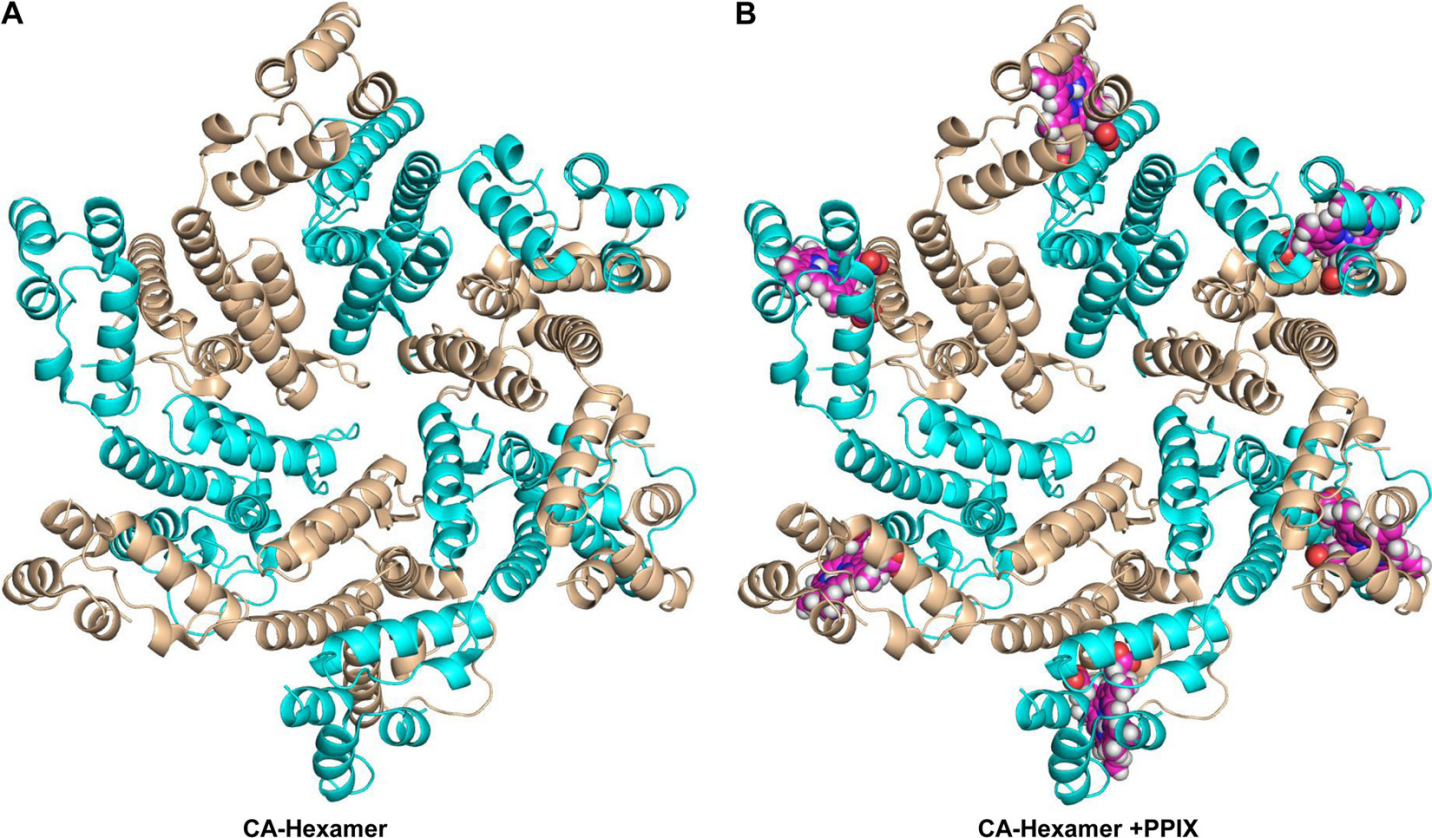
PPIX binds to the pockets of hexameric CA. (A) The crystal structure of hexameric CA (PDB ID: 4XRO). (B) The CA hexamer with docking poses of PPIX, which was made via the superposition of the CA CTD-PPIX complex onto the CA NTD/CA CTD interfaces in hexameric CA. The models of hexameric CA were created via crystallographic symmetry. The capsid is shown as a cartoon, with each protomer colored in either cyan or wheat. The atoms comprising PPIX are shown as spheres (C in magenta, H in white, N in blue, and O in red). All molecular graphics were generated using PyMOL.

**FIG 7:**
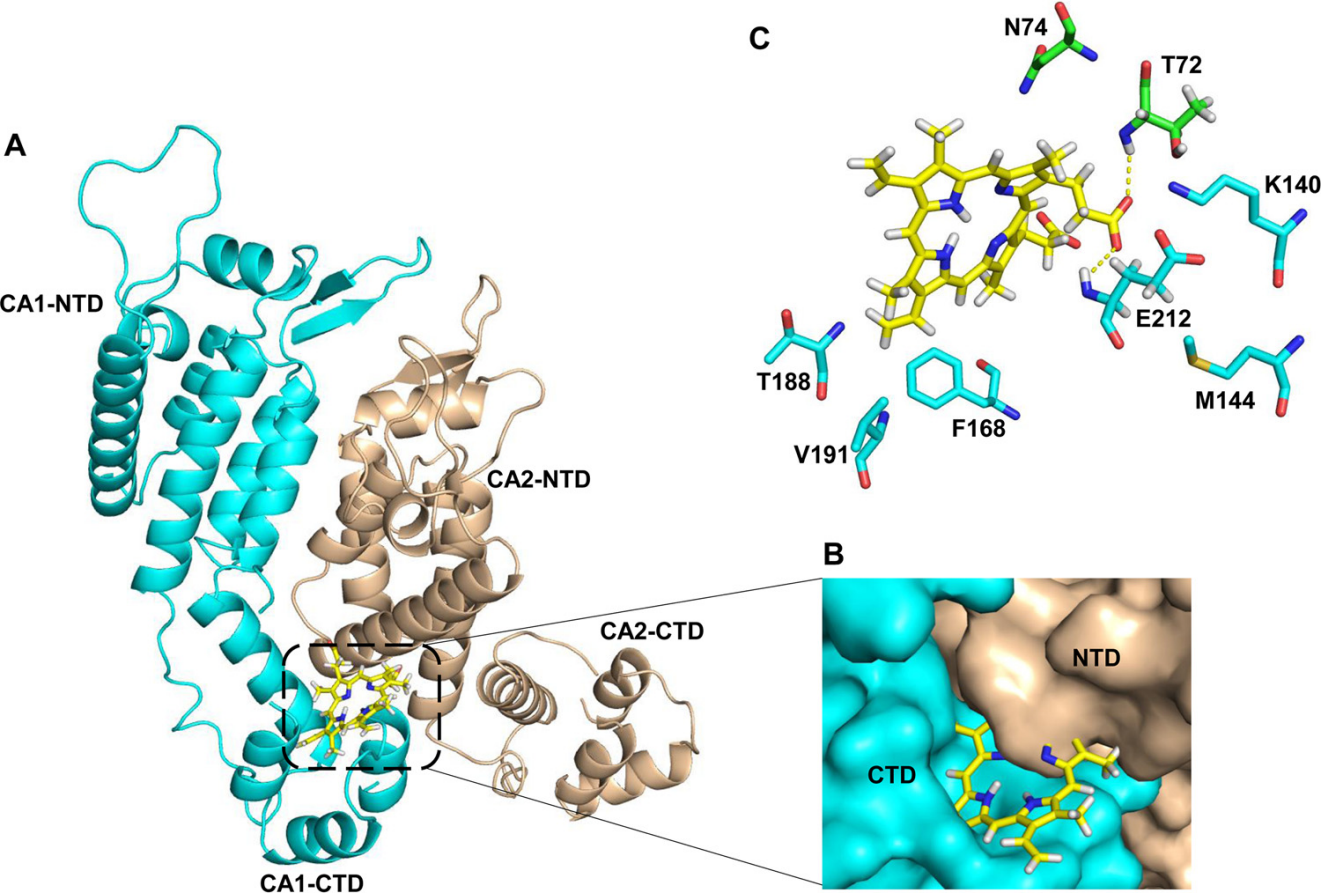
Binding mode of PPIX to the CA NTD/CACTD interface of the CA dimer. (A) The complex structure of the CA dimer (PDB: 4XRO) with PPIX. The pocket is formed by helices 3 and 4 of CA NTD (cyan) and by helices 8 and 9 of the adjacent CA CTD (wheat). The CA dimer was extracted from the CA hexamer (PDB: 4XRO). (B) Closeup view of PPIX in its binding pocket. The adjacent CA NTD and CA CTD from two protomers are colored in wheat and cyan, respectively. (C) Key residues providing key interactions with PPIX. PPIX forms H-bonds by contacting residue T72 in one protomer and E212 in one adjacent protomer. All molecular graphics were generated using PyMOL.

To characterizing the binding of PPIX to the CA hexamer, a CA hexamer structure with or without PPIX was analyzed based on two crystal structures (PDB IDs: 2BUO and 4XRO). First, the binding profile of PPIX to the pocket in CA CTD (PDB ID: 2BUO) was predicted via molecular docking using Swiss-Dock and PyMOL. PPIX can bind to CA CTD through H-bonds formed with L211 and E212 (Fig. 5A and B). PPIX was also analyzed via molecular dynamics (MD) simulation to confirm its binding to the pocket in CA CTD. As shown in Fig. S2A. the position of PPIX is similar to the binding pose. The distance between the carboxylate oxygen of PPIX and the hydrogen from the backbone of L211 as well as the distance between the carboxylate oxygen of PPIX and the hydrogen from the backbone of E212 were computed from the MD simulation. As shown in the distance changes along the simulation time (Fig. S2B), the carboxylate group of PPIX can shift between L211 and E212 to form hydrogen bonds. The MD simulation suggested that PPIX can bind stably with CA CTD. Then, this CAI CTD-PPIX complex was superimposed over the crystal structure of hexameric CA (PDB ID: 4XRO) (Fig. 6A). The results of the superposition revealed that PPIX can bind to the interface formed by adjacent protomers of the CA hexamer (Fig. 6B). Moreover, the PPIX binding profile was further revealed via the superposition of the CAI CTD-PPIX complex over the pocket of the dimeric CA extracted from the hexameric CA. The results showed that the PPIX molecule can bind at the interfaces between CA subunits (Fig. 7A and B), without affecting the paired protomers, via the E212 and T72 residues. Taken together, these results suggested that PPIX can preferentially bind to the pockets of hexameric CA.

## DISCUSSION

HIV-1 CA CTD is essential for viral particle assembly (35, 36). The CAI peptide, which leads to an allosteric destabilization of the CA dimer interface by binding to a conserved hydrophobic pocket of CA CTD, disrupts the *in vitro* assembly of spherical and tubular particles (21, 30). Therefore, this allosteric site in the CA CTD can be considered as a target for antiviral therapy. We have previously developed and validated an HTRF-based assay for the screening of inhibitors that target the CAI-binding pocket. Using this method, three compounds were identified as binding to this cavity and demonstrated their inhibitory effects against HIV-1 replication, thereby validating the efficiency of the developed HTRF assay in identifying the assembly inhibitors (37, 38). In the present study, we used the developed assay to screen a library in order to identify compounds that may interact with the CAI-binding pocket in CTD. In the present study, protoporphyrin IX (PPIX) was identified as a promising lead candidate through a combination of a developed screening assay, secondary assays, CA assembly assays, and a BLI assay.

Target engagement is needed to validate the interaction of the primary hits with their targets in drug screening campaigns (39, 40). In this study, 5 compounds (thioctic acid, riboflavin, stictic acid, protoporphyrin, and theaflavin) were identified as primary hits, all of which competed with the CAI peptide to bind to CA CTD. However, after performing the target engagement to check whether or not these compounds were CA-targeting compounds by using the BLI assay, only PPIX showed binding affinity to CA. Three other compounds (riboflavin, stictic acid, and theaflavin) appeared as nonspecific inhibitors of the CA CTD-CAI interaction, which could be caused by their interference with the HTRF screening system or with the aggregation of CA.

To date, several small molecules and peptides have been demonstrated to bind to CA and to suppress viral replication, but they have distinct effects on capsid assembly. Some compounds decrease CA assembly, which is demonstrated by CAI and BI64 (BM1), whereaothers accelerate CA assembly, which is demonstrated by PF74 and GS-CA1 (12). It is proposed that the different modes of action of CA assembly modulators can be caused by their different affinities for the CA monomer and hexamer (41). To characterize the effects of previously described CA-targeting compounds on CA assembly, we categorized several of these compounds, based on their affinity to the CA hexamer, into groups A and B, (Table 2). For PF74 and GS-CA1 (group A), the structural data and a biophysical analysis indicated that both compounds preferentially bind to the assembled CA hexamer rather than the CA monomer (42, 43). These two compounds act like a glue and stabilize the CA CTD-NTD intersubunit contacts, which is indispensable for the formation of assembled CA and leads to a stable but aberrant CA structure (44). In contrast, CAI and BM2 were observed to bind to the CA monomer and showed no detectable binding to the CA hexamer (38, 45). Moreover, the structural information revealed that their binding to CA would disrupt the interface contacts that are required for the assembly of CA (21, 30, 31). Our data demonstrated that PPIX had higher affinities to the hexamer than to the monomer, and the molecular docking analysis mapped the binding site of PPIX to the CA NTD/CACTD interface of assembled CA, which can together explain the effects of PPIX on CA assembly (i.e., increasing multimerization) and are consistent with the findings of previous studies (38).

**TABLE 2:** Profiles of PPIX and the other CA inhibitors

Group	Modulation of CM^*a*^	Compound	Binding affinity (μM)		Reference
			Monomeric CA	Hexameric CA	
A	Increase	PF74	1.34	0.021	42
		6a-9	7.95	6.04	52
		GS-CA1	3.9	0.39	40
		H22	ND	38.2	53
		GS-6207	2.5	0.24	14
		PPIX	4.3	0.53	
B	Decrease	CAI	1.38	ND^*b*^	38
		BM2	0.14	ND	38

a CM: CA multimerization.

b ND: Not detected.

PPIX, the final intermediate for heme biosynthesis (46), was used for cancer diagnosis (47) and as a photosensitizer with which to treat cancer (48, 49). It has been well-documented in the literature that PPIX exerts broad-spectrum antiviral activity by suppressing several enveloped viruses, including HIV-1, influenza A, vesicular stomatitis virus, severe acute respiratory syndrome coronavirus 2, Lassa virus, and Machupo virus (50–52). In these previous investigations, the antiviral mechanism of PPIX was reported as the inhibition of the entry of viruses. In terms of HIV-1, PPIX prevents viral infection by blocking the binding of the V3 loop of the envelope glycoprotein gp120 to CD4 (53, 54). In the present investigation, PPIX was identified as a modulator of HIV-1 CA multimerization. PPIX binds to the interface formed by the NTD and the CTD between adjacent protomers in the CA hexamer via the T72 and E212 residues, serving as a glue to enhance the multimerization of CA. It possesses a structure containing a porphyrin core with two aliphatic carboxy groups, which is different from those of the previously described CA inhibitors (55, 56). Although we are not sure whether or not PPIX is a capsid inhibitor *in vivo*, which is a limitation of this study and requires further study, PPIX could be used as a new lead for the design of HIV-1 CA assembly modulators.

### Conclusion.

In the present study, we identified PPIX as an HIV-1 CA multimerization modulator that increases CA assembly by targeting the assembled CA hexamer. As far as we know, the structure of PPIX, is different from those of the previously described CA modulators, although PPIX is not a new compound. In the future, other derivatives of porphyrin should be tested in order to evaluate their CA assembly modulation activity. Additionally, the structure of PPIX in complex with the capsid hexamer should be determined in order to reveal its binding site in CA. In conclusion, our study demonstrated that PPIX is a potent CA assembly modulator and throws new light on the development of novel structural anti-HIV-1 inhibitors.

## MATERIALS AND METHODS

### Agents and inhibitor libraries.

The Spectrum Collection Library was provided as 10 mM stock solutions in dimethyl sulfoxide (DMSO) by the National Compound Resource Center (Shanghai, China). Anti-GST-XL665, streptavidin-europium cryptate, and white shallow well microplates (384-well) were obtained from PerkinElmer (Boston, MA). Black 96-well microplates were purchased from Greiner Bio-One (Darmstadt, Germany). Streptavidin-coated BLI biosensors were provided by Sartorius AG (Goettingen, Germany). The CAI-biotin peptide (ITFEDLLDYYPGGGSK-biotin) was synthesized at GL Biochem (Shanghai) Ltd. PF74 was commercially available from the supplier MedChem Express (Shanghai, China). Ni-nitriloacetic acid agarose resin and glutathione agarose beads were purchased from Smart-Lifesciences (Changzhou, China). Other reagents were obtained from AMRESCO (Solon, USA).

### Recombinant CA overproduction and purification.

Recombinant wild-type (WT) and mutant HIV-1_HXB2_ Cas were produced using a previously described method (57). The C-terminal domain of CA, expressed as an N-terminal glutathione *S*-transferase (GST) fusion protein, was purified as previously described (25, 37). CA hexamers were obtained by introducing quadruple mutations (A14C/E45C/W184A/M185A) as previously described (58). The purification of the mutant CA protein was performed following the same procedure as for the wild-type CA protein. Aliquots of the purified protein were flash-frozen in liquid nitrogen and stored at −80°C.

### Primary screening.

For the primary screening, each compound (10 mM in stock) was diluted to 250 μM in reaction buffer (phosphate-buffered saline supplemented with 2 mM β-mercaptoethanol and 0.05% Tween 20). The protein and the peptide were also diluted in the reaction buffer. To perform the primary screening, a previously developed method was used (37). Briefly, one micromole of each compound (i.e., 2 μL CAI-biotin peptide and 2 μL GST-CA CTD) were dispensed into 384-well white shallow microplates. This was followed by incubation at room temperature (RT) for 30 min. Then, 5 μL of premixed HTRF agents (fluorescent donor and acceptor) were added. The plates were incubated for an additional 1 h at RT, and the HTRF signal for each well was acquired by reading the plates at 665 nm and 620 nm in a PerkinElmer Envision Multilabel Plate Reader. The data analysis and visualization were performed using GraphPad Prism 5.0. All of the compounds were assayed in duplicate to ascertain reproducibility. Each plate was analyzed and passed quality control (QC) if its Z′-factor was greater than 0.5. The Z′-factor that was used to estimate the assay quality was calculated using the following equation: Z' = 1 − (3 × SD_max_ + 3 × SD_min_) / (|μ_max_ − μ_min_|), where SD_max_ and SD_min_ are the standard deviations of the positive-control and negative-control measurements, respectively, and μ_max_ and μ_min_ are the means of the respective positive and negative signal controls, respectively (59).

### Dose-response curves determined for hits from the primary screening.

Compounds showing an inhibitory rate of greater than 70 percent in the primary screening were repurchased and reassessed in dose-response studies with 2-fold serial dilutions from 50 μM to 0.39 μM. The dose-response assays were carried out in triplicate. The half-maximal inhibitory concentration (IC_50_) for each compound was determined by the fitting of experimental data using GraphPad Prism (version 5.0).

### *In vitro* HIV-1 CA assembly assay.

A previously described method (19, 57, 60) with some modifications was used to monitor the effect of PPIX on HIV-1 CA assembly. Prior to the assay, purified CA was dialyzed against 50 mM sodium phosphate at pH 8.0 and was concentrated to at least 120 μM. One micromole of compound (5 mM) was added to a 74 μL assay buffer that was made by mixing NaCl (5 M) with NaH_2_PO_4_ (pH 8.0, 200 mM) in a 2:1 ratio by volume. To start the assay, 25 μL of prepared CA were added. DMSO was used as a vehicle control. The samples were equilibrated for 2 min prior to their measurement. The absorbance values at 350 nm for each sample were acquired every 30 s for 20 min using an EnVision multimode plate reader (PerkinElmer). The raw data were corrected for the absorbance values of a sample without NaCl.

### Biolayer interferometry assay.

In a biolayer interferometry (BLI) assay, a biomolecular bait is immobilized on a matrix at the tip of a fiber-optic sensor. The binding between the immobilized ligand and another molecule in an analyte solution produces a change in the optical thickness at the tip and results in a wavelength shift that is proportional to the binding. BLI provides direct binding affinities as well as rates of association and dissociation (61). The CA monomer and hexamer were labeled with biotin using EZ-Link-Sulfo-NHS-Biotin. *In vitro* CA binding was confirmed via a BLI assay that was performed on an Octet96 at 25°C. Each sample (200 μL) was dispensed to a black 96-well plate. Streptavidin biosensor (SA) tips were pre-wet for at least 10 min in assay buffer (0.02% Tween 20 and 1% DMSO in phosphate-buffered saline, pH 7.5) prior to use. The biotinylated protein was immobilized onto SA biosensors by exposing the biosensor tips in polymerase chain reaction (PCR) tubes that contained 15 μL of protein (50 μg/mL) for 5 min at RT. Sensors for background reference subtraction were coated without the biotinylated protein. Kinetic measurements were taken by dipping the CA-coated SA sensor in assay buffer for 60 s, and then transferring it into wells containing compound for 120 s of association followed by redip in the assay buffer for 120 s of dissociation. The raw data were processed via double reference subtraction to remove signals caused by solvent effects and nonspecific binding. Then, Sensorgrams were fit using OctetRed user software (version 9.2) with a global 1:1 model to obtain the values of three constants: k_on_, k_off_ and K_D_.

### Molecular docking.

The crystal structures of CA CTD (PDB ID: 2BUO) and the CA hexamer (PDB ID: 4XRO) from the Protein Data Bank were utilized for the molecular docking. All docking simulations were done using the “Swiss Dock” server (62, 63). The figures were generated using the PyMol molecular visualization software package (http://www.pymol.org) or the UCSF Chimera software package (64).

### Data availability.

The data that support the findings of this study are openly available in Mendeley Data (65).
