# Supplementary material for: A Broad-Spectrum Antiviral Molecule, Protoporphyrin IX, Acts as a Moderator of HIV-1 Capsid Assembly by Targeting the Capsid Hexamer
Source: Microbiol Spectr. 2022 Dec 7;11(1):e02663-22. doi: 10.1128/spectrum.02663-22 (PMC9927277; doi:10.1128/spectrum.02663-22)
Supplement: Supplemental file 3 — Supplemental material. Download spectrum.02663-22-s0003.pdf, PDF file, 0.2 MB [file spectrum.02663-22-s0003.pdf]

Supplementary materials

Method

Molecular dynamics

The docking pose was examined by molecular dynamics (MD) simulation. The force field parameters for ligand PPIX were obtained from General AMBER force field (GAFF) [1] using Antechamber module of AmberTools. AM1-BCC charges were assigned to the atoms of ligand. The generalized parameter file of ligand was converted into GROMACS format by acpype.py script [2]. The protein was described by AMBER99SB-ILDN force field and water model is described by TIP3P model. The complex conformation obtained from docking described above was dissolved in a water box with distance 12 Å from the protein to the box boundary. The complex structure was minimized by steepest descent algorithm. Then the complex was equilibrated by 500-ps NVT using the Berendsen thermostat (coupling constant: 0.1 ps), and 500-ps NPT simulations using the V-rescale thermostat (coupling constant: 0.1 ps) and Parrinello-Rahman barostat (coupling constant: 2.0 ps). We then carried out 20-ns production MD simulations. Both the van der Waals interactions and short-range electrostatic interactions were calculated using a cutoff of 12 Å, and the long-range electrostatic interactions are computed using the PME method. All MD simulations were performed by GROMACS software package [3].

Table titles

Table S1 Compound information.

Table S2 Drug screening results.

Table S1 and S2 are available at https://data.mendeley.com/datasets/vvtfdgfk76.

Figure S1 The responses of the compounds screened at a single concentration of 30 μM. PPIX with response over than 0.03 nm was labeled red.

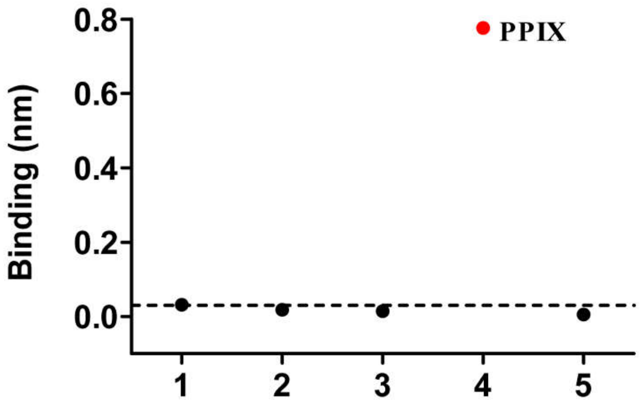

Figure S2 PPIX was analyzed by MD simulation to find its binding to the pocket in CA-CTD.

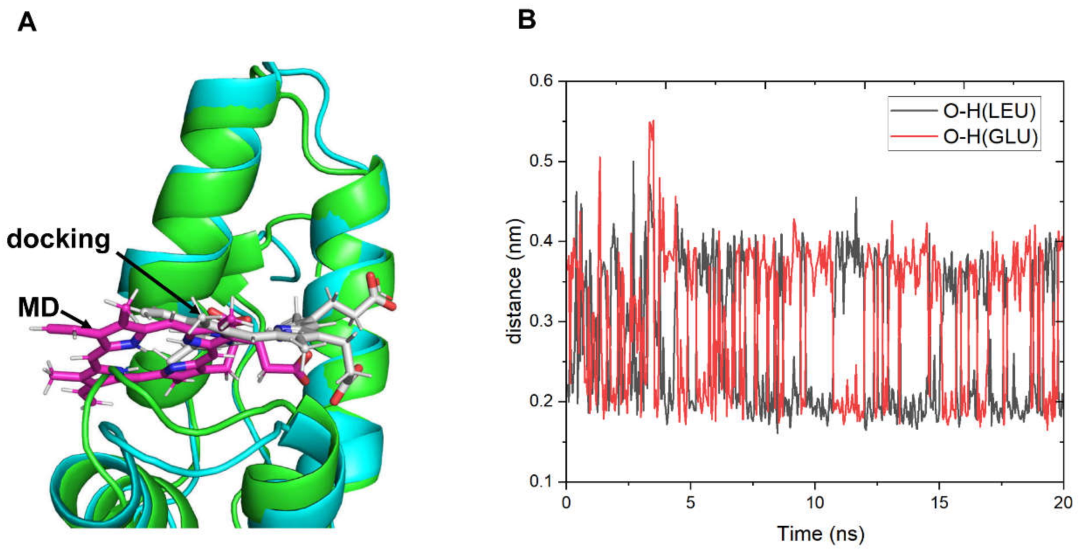

Reference

1. Wang J, Wolf RM, Caldwell JW, Kollman PA, Case DA: Development and testing of a general amber force field. Journal of Computational Chemistry 2004, 25(9):1157-1174.
2. Sousa da Silva AW, Vranken WF: ACPYPE - AnteChamber PYthon Parser interfacE. BMC Research Notes 2012, 5(1):367.
3. Van Der Spoel D, Lindahl E, Hess B, Groenhof G, Mark AE, Berendsen HJC: GROMACS: Fast, flexible, and free. Journal of Computational Chemistry 2005, 26(16):1701-1718.
